# Airborne Fumigants and Residual Chemicals in Shipping Containers Arriving in New Zealand

**DOI:** 10.1093/annweh/wxab090

**Published:** 2021-10-18

**Authors:** Ruth Hinz, Andrea ’t Mannetje, Bill Glass, Dave McLean, Jeroen Douwes

**Affiliations:** Centre for Public Health Research, Massey University, Wellington, New Zealand; Centre for Public Health Research, Massey University, Wellington, New Zealand; Centre for Public Health Research, Massey University, Wellington, New Zealand; Centre for Public Health Research, Massey University, Wellington, New Zealand; Centre for Public Health Research, Massey University, Wellington, New Zealand

**Keywords:** exposure estimation, occupational groups, shipping container/sea container, fumigation/fumigant, volatile organic compounds, workplace exposure standard, occupational exposure

## Abstract

**Background:**

Airborne fumigants and other hazardous chemicals inside unopened shipping containers may pose a risk to workers handling containers.

**Methods:**

Grab air samples from 490 sealed containers arriving in New Zealand were analysed for fumigants and other hazardous chemicals. We also collected grab air samples of 46 containers immediately upon opening and measured the total concentration of volatile organic compounds in real-time during ventilation. Additive Mixture Values (AMV) were calculated using the New Zealand Workplace Exposure standard (WES) and ACGIH Threshold Limit Values (TLV) of the 8-h, time-weighted average (TWA) exposure limit. Regression analyses assessed associations with container characteristics.

**Results:**

Fumigants were detectable in 11.4% of sealed containers, with ethylene oxide detected most frequently (4.7%), followed by methyl bromide (3.5%). Other chemicals, mainly formaldehyde, were detected more frequently (84.7%). Fumigants and other chemicals exceeded the WES/TLV in 6.7%/7.8%, and 7.8%/20.0% of all containers, respectively. Correspondingly, they more frequently exceeded ‘1’ for the AMV-TLV compared to the AMV-WES (25.7% versus 7.8%). In samples taken upon opening of doors, fumigants were detected in both fumigated and non-fumigated containers, but detection frequencies and exceedances of the WES, TLV, and AMVs were generally higher in fumigated containers. Detection frequencies for other chemicals were similar in fumigated and non-fumigated containers, and only formaldehyde exceeded both the WES and TLV in both container groups. Volatile compounds in container air reduced rapidly during ventilation. Some cargo types (tyres; personal hygiene, beauty and medical products; stone and ceramics; metal and glass; and pet food) and countries of origin (China) were associated with elevated airborne chemical and fumigant concentrations.

**Conclusion:**

Airborne chemicals in sealed containers frequently exceed exposure limits, both in fumigated and non-fumigated containers, and may contribute to short-term peak exposures of workers unloading or inspecting containers.

**What’s Important About This Paper?**

This is the first study that comprehensively assessed airborne concentrations of fumigants and other chemicals in containers imported into New Zealand. Contaminant concentrations in sealed fumigated and non-fumigated containers frequently exceeded exposure limits, putting workers at risk when opening container doors, but it remains difficult to predict which containers represent the greatest risk of high exposure. There is a need to establish improved and standardized strategies for the safe inspection and unpacking of shipping containers.

## Introduction

Globally, shipping container throughput has risen from 622 to 802 million twenty-foot equivalent units (TEU) between 2012 and 2019 (Statista, 2020). A proportion of containers requires fumigation either for biosecurity reasons or to prevent damage to the cargo. Commonly used fumigants include phosphine (hydrogen phosphide), methyl bromide, and ethylene oxide (OSH WIKI, 2018), which are toxic to both pets and humans.

Sealed shipping containers allow for only limited natural ventilation during transport (Svedberg and Johanson, 2017); fumigants and off-gassed chemicals (e.g. formaldehyde, toluene, and benzene) from cargo or packaging may therefore accumulate in the air, potentially reaching unsafe levels (European Agency for Safety and Health at Work, 2018). High levels of airborne chemicals have been found in sealed containers, and several acute poisonings in workers handling shipping cargo have been reported (Spijkerboer *et al.*, 2008; Breeman, 2009; Verschoor *et al.*, 2010, 2011; Preisser *et al.*, 2011, 2012; Budnik *et al.*, 2012; Kloth *et al.*, 2014; Roberts *et al.*, 2014; Baur *et al.*, 2015; European Agency for Safety and Health at Work, 2018), with symptoms ranging from skin irritation to severe respiratory distress and persistent neurological deficits.

Personal exposures to fumigants and off-gassed chemicals measured in container workers are generally lower than levels measured in sealed containers (Hinz *et al.*, 2020), likely due to the rapid decline in concentration following the opening of containers and subsequent ventilation (Svedberg and Johanson, 2013; Braconnier and Keller, 2015). Nonetheless, high exposure may still occur (Svedberg and Johanson, 2013), particularly upon opening of container doors (Svedberg and Johanson, 2013; Braconnier and Keller, 2015).

Although several factors may affect chemical concentrations in shipping containers such as cargo, packaging materials, and number of vents (Knol-de Vos, 2002, 2005; Baur *et al.*, 2010; Bethke *et al.*, 2013; Svedberg and Johanson, 2013; Braconnier and Keller, 2015; Johanson and Svedberg, 2020), it is difficult to identify which containers may pose a health risk to workers, mainly due to a lack of suitable and affordable devices to measure exposure (Svedberg and Johanson, 2017; European Agency for Safety and Health at Work, 2018).

Annually, approximately 500,000 import containers arrive in New Zealand by sea (New Zealand Ministry of Transport, 2019) with some always being fumigated (for specific types of cargo) and others being fumigated only in particular seasons (e.g. during marmorated stink bug season) or when biosecurity intrusions are detected. In this study, we assessed fumigant and residual chemical concentrations inside sealed containers imported into New Zealand and upon opening of container doors. We also assessed associations with cargo and container characteristics and compared chemical concentrations inside sealed containers with international findings.

## Methods

### Study design

This study involved measurements of airborne chemicals in shipping containers arriving in New Zealand and consisted of two parts: (i) a survey of chemical concentrations in 519 sealed containers; and (ii) a smaller, more detailed, survey of concentrations measured upon opening of 46 containers. Sample sizes were based on practical considerations balancing research funding, minimizing disruption for workers, and the need to have enough samples to draw conclusions.

The larger survey, conducted between February and June 2011, involved a random sample of imported containers arriving in the Port of Tauranga in New Zealand, with information collected (from the Customs database) on cargo categories and country of origin (see Table S1 available at *Annals of Occupational Hygiene* online); information on fumigation status and whether the container was full or only partially loaded was also collected. Most containers were loaded directly from the vessels onto rail carriages for further transport. The measurements were taken by Customs officers when containers were on rail carriages, with a small proportion of containers measured at the port customs inspection facility.

The smaller survey, conducted between 2013 and 2016, was nested in a cross-sectional exposure and health study of workers handling cargo from shipping containers and export logs (Hinz *et al.*, 2020). It involved 16 New Zealand Ministry of Primary Industries Accredited Transitional Facilities (ATF) that open and inspect overseas containers. The cargo and country of origin of the containers differed widely (Table S2 available at *Annals of Occupational Hygiene* online) and included containers previously fumigated overseas and/or in New Zealand. Management provided permission for sampling and identified suitable containers based on workplace requirements as well as an interest in specific containers (e.g. when they gave off chemical odours or were previously fumigated), leading to an oversampling of fumigated containers. Information was collected on fumigation status from signage on the container, and/or shipping documentation obtained from container handlers and management. Information on cargo category, country of origin, container size, number of open container vents, whether cargo was on pallets, temperature in the container and barometric pressure, was based on observations by research staff and/or provided by container handlers and management.

### Sampling

For the larger survey, grab air samples (samples taken for ≤1 s) were collected using a probe penetrated between the rubber seals at the bottom of the container door to a depth of 10–25cm. Air was collected into a Tedlar^®^ bag and analysed (see details below) immediately on site (New Zealand Customs Service, 2012).

**Figure 1.**
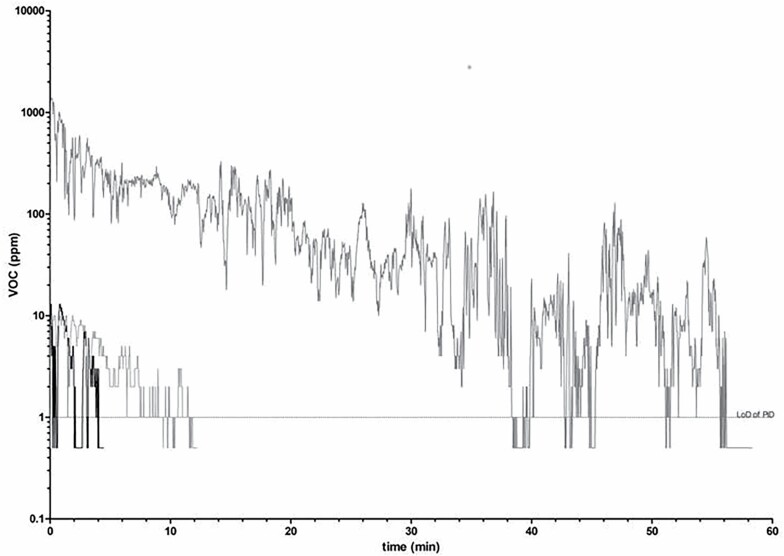
PID readings (ppm) upon opening containers. Data shown for three containers that had PID readings of >1 ppm for >2 min.

For the smaller survey, we took grab air samples at medium height (1.5–1.7 m) at the entrance of the container immediately when the doors were opened (most containers were positioned outdoors). Samples were taken by connecting a Teflon tube to a 400cc stainless steel and Siltek treated sampling canister (Restek Corporation, PA, USA) negatively pressurized to near full vacuum (i.e. ~0 mmHg) by opening the canister valve. Samples were sent to an external laboratory (see below) for analyses. At the same time of collecting grab air samples, we also used a Velocicalc 9565-P/985 photoionization detector (PID) (TSI, Inc., MN, USA) fitted with a 10.6 electron-volt lamp (Ion Science LTD., Cambridge, UK), with a working range of 1000–2000,000 ppb at a temperature range of −10 to 60°C with an accuracy ±0.5°C and a resolution of 1000 ppb, to measure, in real-time, the total concentration of volatile organic compounds (TVOC). The PID was placed on the cargo mainly in the front row at a height of 1.5–2.5 m. If the initial PID reading was above the lower limit of detection (LoD, 1000 ppb), we continued to record until the TVOC concentration fell below the LoD. Readings above 1000 ppb for >2 min were displayed in a graph (Figure 1) and zero readings were given the value 0.5 ppb to be able to display them on a log-scale.

### Laboratory analysis

Samples were analysed using Selected Ion Flow Tube Mass Spectrometry (Syft-Technology, 2005; Milligan *et al.*, 2007; Smith and Španěl, 2015), which provides instant results, is relatively affordable, and allows simultaneous analyses of multiple compounds. However, there is an upper limit to the total amount of reactive compound that can be introduced to the instrument, which, although not a major issue for this study (see Results), is a disadvantage of this method.

Samples from the larger survey were analysed on-site by research staff within an hour of collection using the Voice 100 (Syft Technologies Ltd., Christchurch, New Zealand) for the following chemicals (CAS numbers in brackets): fumigants: 1,2-dibromoethane [106-93-4], chloropicrin [76-06-2], ethylene oxide [75-21-8], hydrogen cyanide [74-90-8], phosphine (hydrogen phosphide) [7803-51-2], methyl bromide [74-83-9]; and for other harmful chemicals frequently detected in containers: benzene [71-43-2], formaldehyde [50-00-0] and toluene [108-88-3]).

Samples from the smaller survey were analysed by Syft Technology using the Voice 200 (Syft Technologies Ltd., Christchurch, New Zealand), an updated version of the Voice 100. Sampling canisters were sent to Syft Technology, with most samples analysed within 24 hours and none later than 48 hours. In addition to the fumigants and other chemicals also analysed in the larger survey (see above), the following chemicals frequently detected in containers were measured: 1,2-dichloroethane [107-06-2], C2-alkylbenzenes [108-38-3, 95-47-6, 106-42-3, 100-41-4], acetaldehyde [75-07-0], ammonia [7664-41-7], methanol [67-56-1] and styrene [100-42-5]. Methanol, although not a chemical of concern to human health in this context, was included later (for 35 of 46 samples) because it has been found frequently at high levels in container air and could be a significant contributor to high PID readings (Svedberg and Johanson, 2017). Blank canisters (field blanks) were analysed with each analysis series, which returned results in the trace-range level only.

As canister samples were stored up to 24 hours, and in some cases up to 48 hours, prior to analysis, we conducted an experiment to assess the stability of several chemicals (formaldehyde, methyl bromide, and benzene) in canisters. This showed an average reduction of 14% (formaldehyde), 6% (methyl bromide), and 10% (benzene) when stored for 24 hours, and 22%, 10% and 22%, respectively, when stored for 48 hours, suggesting that decay was generally modest.

LoDs were calculated from laboratory blanks as follows: LoD = blank result + 3*standard deviation. The LoDs were comparable or somewhat lower for most chemicals in the second survey using the updated Syft Technology Voice 200 device, with the exception of formaldehyde for which the LoD was higher (25 ppb versus 14 ppb; see Tables in Results section for the LoDs of all tested chemicals).

Concentrations were expressed in parts per billion (ppb). In addition to reporting levels for each chemical, we also calculated the additive mixture value (AMV), an estimate of the combined toxic effect of chemicals. To calculate the AMV, each chemical was given a toxicity score by dividing the measured level by the exposure standard, followed by the summation of the toxicity scores of all chemicals measured in the sample. The ‘AMV-TLV’ $(\overset{}{\sum }C_{i}/TLV_{i},\;\;C=concentration,\;i=number\;of\;chemicals)$ was based on the American Conference of Governmental Industrial Hygienists Threshold Limit Values (TLV) (ACGIH, 2020); the ‘AMV-WES’ $\left(\sum C_{i}/WES_{i}\right)$ was based on the New Zealand Workplace Exposure standards (WES) (Worksafe New Zealand, 2020). An AMV exceeding ‘1’ was considered to be above the exposure limit for that mixture. For the calculation of AMVs, measurements below the LoD were assigned a value of half the LoD. We also calculated AMVs separately for fumigants and other chemicals (non-fumigants), and we calculated AMVs including and excluding methanol. We excluded 1,2-dibromoethane from the calculation of AMVs because the ACGIH did not specify a TLV or ceiling limit and the New Zealand WES is below the LoD of 1,2-dibromoethane.

### Statistical analyses

Analyses were conducted using Stata version 15.1 (StataCorp LP, Texas, USA). Medians, 25–75 percentiles and maximum levels were used to summarize chemical concentrations, with samples with concentrations below the lower limit of detection or above the upper limit of quantification excluded.

Associations between container characteristics and chemical concentrations and/or AMVs were initially assessed using univariate linear regression (data not shown); variables that showed statistically significant associations were subsequently tested in multivariable analyses (mutually adjusting for other co-variables). To assess associations with cargo and country of origin, cargo was grouped into 14 categories (see supplementary Table 1) and country into six groups: Australia, China, North America, Europe, other Asian countries, and ‘other regions’. Concentrations were ln-transformed; regression coefficients were therefore expressed as a relative difference or ratio (calculated as *e*^(regression coefficient)^), with, for example, a ratio of 2 indicating that the AMV for a particular cargo category was two times higher compared to the reference category, while a ratio of, for example, 0.7 indicates a 30% lower AMV level. Due to the high number of concentrations below the lower LoD we used left-censored regression (Tobit). Reference groups were chosen such that they represented a sufficient sized group and were characterized by relatively low chemical concentrations, hence they vary between both surveys. Analyses were repeated for AMVs calculated for fumigants only, or other chemicals only.

We conducted similar analyses for the smaller survey, but with additional variables: fumigation status (yes/no), container size (20 ft/40 ft), and number of container vents (0/2/4). We also applied linear regression to assess associations with fumigation status and concentrations of individual chemicals. Concentrations of individual chemicals were ln-transformed, and we used left-censored (at the LoD level) regression (Tobit) due to the high number of measurements below the lower LoD.

## Results

In the larger survey, 26 samples were excluded due to containers not being able to be linked to Customs data, being empty, or having arrived from an intermediate destination, leaving 493 samples for analyses. From these, three had levels above the upper limit of quantification and were excluded because no value could be allocated to any of the chemicals tested. Only two containers had been identified as fumigated. For the smaller survey, 46 samples were available, 29 from non-fumigated and 17 from fumigated containers. TVOCs measurements were available for 41 containers.

### Samples taken from sealed containers

Fumigants were detectable in 11.4% of containers (Table 1), with ethylene oxide detected most frequently (4.7%), followed by methyl bromide (3.5%). Other chemicals were detected more frequently (84.7%), with the highest detection rate for formaldehyde (81%, Table 1). Levels of fumigants and other chemicals exceeded the WES in 6.7% and 7.8% of all containers, respectively. As the WES for 1,2-dibromoethane is below the LoD, the proportion of containers in which fumigant levels exceeded the WES is likely higher. Levels exceeding the TLV were more common (7.8% and 20.0% of containers for fumigants and other chemicals, respectively; Table 1). Correspondingly, compared to the AMV-WES, the AMV-TLV more frequently exceeded ‘1’ (25.7% versus 7.8%).

**Table 1.** Descriptive statistics (medians are based on samples with levels >LoD) of sealed container air samples (*n* = 490)

Variable (ppb^a^)	WES^b^	TLV^c^	LoD^d^	>LoD (*n*/%)	Median# (p25-p75)	maximum	>WES (%)	>TLV (%)
Fumigants				56/11.4			6.7	7.8
1,2-Dibromoethane	0.3	n/a^e^	13	10/2.0	239.2 (66.5–693.9)	1066.1	> 2.0^f^	n/a
Chloropicrin	100	100	20	1/0.2	50.2	50.2	0	0
Ethylene oxide	100	1,000	10	23/4.7	1241.5 (689.6–1922.2)	9717.02	4.7	2.9
Hydrogen cyanide	10 0000^g^	4700^g^	2	9/1.8	232.7 (132.4–448.2)	539.54	0	0
Phosphine	300	50	5	5/1.0	51.5 (20.0–82.9)	144.03	0	0.6
Methyl bromide	5000	1000	13	17/3.5	415.3 (78.6–2,734.7)	49 890.9	0.2	1.2
Other chemicals				415/84.7			7.8	20.0
Benzene	1000	500	10	17/3.5	210.0 (112.0–299.0)	3069.9	0.2	0.6
Formaldehyde	500	100	14	397/81.0	45.3 (26.4–87.6)	6562.0	2.9	18.0
Toluene	20 000	20 000	10	156/31.8	115.5 (61.2–244.1)	6840.7	0	0
Overall				416/84.9			7.8	20.2
Cumulative							AMV >1 (%)	AMV >1 (%)
AMV - WES	1	1	n/a	n/a	0.2 (0.2–0.4)	97.9	7.8	–
AMV - TLV	1	1	n/a	n/a	0.6 (0.4–1.0)	70.3	–	25.7

n/a. not applicable.

^#^Based on samples with concentrations >LoD (i.e. samples with concentrations <LoD were not included).

^a^ppb: parts per billion.

^b^8-h workplace exposure standards (WES) set by Worksafe New Zealand (2020).

^c^8-h workplace exposure standards (TLV-Threshold Limit Value) set by American Conference of Governmental Industrial Hygienists (ACGIH) (2020).

^d^Limit of detection.

^e^The ACIGH has not set a TLV for 1,2-dibromoethane.

^f^For 1,2-dibromoethane, the number of occurrences exceeding the WES could be higher as its WES is below its LoD.

^g^These chemicals do not have a TWA limit but only a ceiling limit which was used instead.

The cargo category ‘rubber products including tyres’ was associated with higher AMVs compared to the reference category, for both fumigants (AMV-WES: ratio 4.0, 95% CI 2.2–7.2; AMV-TLV: ratio 2.6, 95% CI 1.8–3.9) and other chemicals (7.6, 4.0–14.7 and 7.9, 4.1–15.6, respectively; Table 2). AMVs (WES and TLV) for fumigants were also positively associated with: ‘personal hygiene, beauty and medical products’, ‘stone, ceramics and articles thereof’, and ‘metal and glass’. AMVs for other chemicals were positively associated with ‘pet food’, due to high formaldehyde levels, and negatively for ‘metal and glass’ (Table 2). Containers from Europe had a higher AMV-WES for fumigants compared to containers from North America (Ratio 1.7, 95% CI 1.0–2.5) even after adjusting for container cargo; containers from China had higher AMVs for other chemicals (ratio 1.7, 95% CI 1.1–2.6 for both AMV-WES and AMV-TLV; Table 2). Table S1, available at *Annals of Occupational Hygiene* online, provides the percentage of detectable fumigants/chemicals in container air for each cargo type and country, showing that detectable levels of fumigants, formaldehyde, and toluene were observed in most cargo and country categories.

**Table 2.** Multi-variate regression of AMVs with cargo category and country of origin, sealed container survey (*n* = 490)

		Fumigants		Other chemicals		Overall	
		AMV-WES^a^	AMV-TLV^b^	AMV-WES	AMV-TLV	AMV-WES	AMV-TLV
Variable	n	Ratio (CI)	Ratio (CI)	Ratio (CI)	Ratio (CI)	Ratio (CI)	Ratio (CI)
Cargo							
Food, beverages, tobacco	78	reference	reference	reference	reference	reference	reference
Personal hygiene, beauty and medical products	42	1.5(1.0–2.1)*	1.3(1.0–1.7)*	1.2(0.8–1.7)	1.2(0.8–1.8)	1.5(1.0–2.1)*	1.3(0.9–1.7)
Plastics	58	1.4(1.0–1.9)	1.2(1.0–1.5)	0.9(0.6–1.3)	0.9(0.6–1.3)	1.3(0.9–1.8)	1.0(0.8–1.4)
Rubber products including tyres	13	4.0(2.2–7.2)***	2.6(1.8–3.9)***	7.6(4.0–14.7)***	7.9(4.1–15.6)***	6.4(3.5–11.6)***	6.5(3.8–11.2)***
Wood and articles there of	20	1.4(0.8–2.3)	1.1(0.8–1.6)	1.1(0.6–1.9)	1.1(0.6–1.9)	1.2(0.7–2.0)	1.0(0.7–1.6)
Paper and paperboard and articles there of	62	1.1(0.8–1.5)	1.0(0.8–1.3)	0.9(0.7–1.4)	1.0(0.7–1.4)	1.0(0.7–1.4)	1.0(0.7–1.3)
Stone, ceramics and articles there of	16	1.8(1.1–3.0)*	1.7(1.2–2.4)**	1.1(0.6–2.0)	1.1(0.6–2.0)	1.6(1.0–2.7)	1.4(0.8–2.2)
Metal and glass	36	1.9(1.3–2.8)**	1.5(1.2–1.9)**	0.6(0.4–0.9)*	0.6(0.4–0.9)**	1.5*(1.0–2.2)	0.9(0.6–1.3)
Machinery, equipment, appliances, electronics	49	1.1(0.8–1.6)	1.0(0.8–1.3)	1.0(0.6–1.4)	1.0(0.6–1.4)	1.0(0.7–1.5)	1.0(0.7–1.4)
Vehicles and part there of	7	1.8(0.9–3.8)	1.2(0.7–2.0)	0.5(0.2–1.2)	0.5(0.2–1.2)	1.3(0.6–2.7)	0.7(0.4–1.4)
Furniture and other man-made fibre articles	32	1.2(0.8–1.9)	1.1(0.8–1.4)	0.9(0.6–1.4)	0.9(0.6–1.4)	1.1(0.7–1.6)	0.9(0.6–1.4)
Pet food	9	1.1(0.6–2.1)	1.0(0.6–1.6)	3.1(1.5–6.5)**	3.3(1.6–7.1)**	1.7(0.9–3.3)	2.4(1.3–4.4)**
Chemicals	36	1.5(1.0–2.2)*	1.2(0.9–1.5)	1.0(0.6–1.5)	1.0(0.6–1.5)	1.4(1.0–2.1)	1.1(0.8–1.5)
Miscellaneous	32	1.1(0.7–1.6)	1.0(0.8–1.3)	1.1(0.7–1.7)	1.1(0.7–1.7)	1.1(0.7–1.7)	1.1(0.7–1.6)
Country							
North America	56	reference	reference	reference	reference	reference	reference
Australia	283	1.1(0.8–1.4)	1.0(0.8–1.2)	0.9(0.6–1.2)	0.9(0.6–1.2)	0.9(0.7–1.3)	0.9(0.7–1.1)
China	74	1.2(0.8–1.7)	1.1(0.9–1.4)	1.7(1.1–2.6)*	1.7(1.1–2.6)*	1.4(1.0–2.0)	1.5(1.1–2.1)*
Asia other than China	33	0.9(0.6–1.4)	1.1(0.8–1.4)	1.2(0.7–1.9)	1.2(0.7–1.9)	1.0(0.6–1.5)	1.1(0.8–1.7)
Europe	21	1.7(1.0–2.8)*	1.3(0.9–1.8)	1.1(0.6–1.8)	1.1(0.6–1.9)	1.6(0.9–2.6)	1.1(0.7–1.8)
Other regions	23	1.6(1.0–2.5)	1.2(0.9–1.7)	1.1(0.6–1.9)	1.1(0.6–1.9)	1.3(0.8–2.2)	1.1(0.7–1.7)

^a^Additive Mixture Value using the WES (8-hour Workplace Exposure Standards set by Worksafe New Zealand (2020)) and excluding 1,2-dibromoethane.

^b^Additive mixture value using the TLV [8-h workplace exposure standards (threshold limit value)] set by American Conference of Governmental Industrial Hygienists (ACGIH) (2020)) and excluding 1,2-dibromoethane.

***P < 0.001, **P < 0.01, *P < 0.05.

### Samples taken upon opening of container doors

Fumigants were detected in both fumigated and non-fumigated containers, but detection frequencies were generally higher in fumigated containers. Similarly, samples collected in fumigated containers more frequently exceeded the WES and TLV for fumigants, except for ethylene oxide and hydrogen cyanide, the latter of which never exceeded the WES or TLV (Table 3). Regression comparing all fumigated and non-fumigated containers (including those with levels below the lower LoD) showed that phosphine, methyl bromide, and ammonia levels were significantly higher (4.7, 78.0, and 53.4 times higher, respectively) in fumigated containers (Table 3). Excluding one container with very high levels of methyl bromide (319,000 ppb) did not change the results (data not shown).

**Table 3.** Concentrations (medians are based on samples with levels >LoD) of fumigants and non-fumigants in containers upon opening of container doors (*n* = 46)

				non-fumigated containers (n=29)^e^					fumigated containers (*n* = 17)^f^					Ratio (95% CI) fumigated versus
Chemical (ppb^a^)	WES^b^	^TLV^ ^c^	LoD^d^	>LoD (n/%)	Median# (p25–p75)	Max	>WES (%)	>TLV (%)	>LoD (n/%)	Median# (p25–p75)	max	>WES (%)	>TLV (%)	non-fumigated containers ^g^
Fumigants														
1,2-dibromoethane	0.3	n/a	5	13/44.8	10.0 (8.0–13.0)	20.1	>44.8^h^	n/a^i^	10/58.8	13.8 (7.2–107.3)	271.8	>58.8^h^	n/a^i^	2.4 (0.9–6.4)
Chloropicrin	100	100	5	7/24.1	10.0 (7.2–25.0)	42.7	0	0	7/41.2	21.9 (6.4–40.4)	193.1	5.9	5.9	2.6 (0.7–9.9)
Ethylene oxide	100	1,000	10	7/24.1	62.6 (37.8–184.0)	2,510.5	6.9	3.4	4/23.5	51.2 (39.4–308.3)	564.5	5.9	0	0.9 (0.1–14.0)
Hydrogen cyanide	10,0000^i^	4,700^j^	3	1/3.4	115.0	115.0	0	0	3/17.6	3.9 (3.0–17.0)	17.0	0	0	8.3 (0.1–554.8)
Phosphine	300	50	3	16/55.2	20.1 (12.7–39.0)	122.0	0	10.3	15/88.2	22.0 (9.7–177.0)	536.3	11.8	29.4	4.7 (1.4–16.0)**
Methyl bromide	5,000	1,000	5	12/41.4	23.4 (7.5–55.6)	352.0	0	0	12/70.6	811.6 (52.5–8,118.1)	319,000.0	17.6	35.3	78.0 (6.0-1020.6)***
Other chemicals														
1,2-dichloroethane	5,000	10,000	5	14/48.3	10.5 (7.6–48.1)	420.4	0	0	10/58.8	30.4 (11.2–73.2)	112.2	0	0	1.9 (0.6–6.5)
C2-alkylbenzenes	50,000	20,000	5	24/82.8	61.0 (17.1–123.8)	978.8	0	0	13/76.5	35.0 (25.7–70.0)	584.7	0	0	0.8 (0.3–2.3)
Acetaldehyde	20,000	25,000^j^	25	25/86.2	143.9 (116.8–453.0)	4,350.0	0	0	15/88.2	201.2 (95.2–534.9)	5,985.1	0	0	1.4 (0.5–3.5)
Ammonia	25,000	25,000	15	3/10.3	93.6 (31.9–330.7)	330.7	0	0	8/47.1	288.5 (117.3–814.7)	1,482.1	0	0	53.4 (2.6-1082.2)**
Benzene	1,000	500	5	7/24.1	18.0 (7.9–37.2)	93.8	0	0	6/35.3	15.9 (11.6–20.6)	161.5	0	0	1.9 (0.4–9.2)
Formaldehyde	500	100	25	22/75.9	187.1 (130.0–248.3)	560.2	3.4	58.6	14/82.4	145.0 (57.6–519.8)	936.6	23.5	47.1	1.1 (0.5–2.5)
Methanol	200,000	200,000	10	25/100.0	1,557.9 (702.4-2,81.0)	25,695.3	0	0	10/100.0	3,435.9 (3,071.5–28,939.0)	72,187.2	0	0	3.2 (0.9–11.9)
Styrene	20,000	10,000	2	10/34.5	3.1 (2.8–15.2)	89.7	0	0	6/35.3	20.3 (7.0–29.3)	702.0	0	0	2.0 (0.3–12.8)
Toluene	50,000	20,000	3	20/69.0	15.7 (7.7–96.3)	459.9	0	0	15/88.2	51.1 (6.9–121.3)	1,559.1	0	0	3.5 (0.9–13.2)
Cumulative							AMV >1 (%)					AMV >1 (%)		
AMV-WES^k^	1	1	n/a	n/a	0.7 (0.2–1.0)	26.4	17.2	-	n/a	1.6 (0.8–2.9)	64.4	52.9	-	3.7 (1.6–8.2)**
AMV-WES fumigants	1	1	n/a	n/a	0.2 (0.1–0.4)	25.3	6.9	-	n/a	0.8 (0.5–2.7)	64.1	41.2	-	5.2(2.2–12.4)***
AMV-WES other chemicals	1	1	n/a	n/a	0.3 (0.1–0.5)	1.2	3.5	-	n/a	0.3 (0.1–1.0)	2.8	23.5	-	1.2 (0.6–2.4)
AMV-TLV^l^	1	1	n/a	n/a	1.6 (0.5–2.9)	8.7	-	69.0	n/a	4.0 (1.8–12.9)	320.5	-	88.2	4.6 (1.9–11.0)***
AMV-TLV fumigants	1	1	n/a	n/a	0.2 (0.1–0.6)	3.0	-	17.2	n/a	3.6 (0.9–7.6)	319.5	-	64.7	12.1 (4.5–32.6)***
AMV-TLV other chemicals	1	1	n/a	n/a	1.5 (0.4–2.3)	5.7	-	62.1	n/a	1.1 (0.3–4.1)	10.4	-	52.9	1.1 (0.5–2.4)

n/a, not applicable.

^#^Based on samples with concentrations >LoD (i.e. samples with concentrations <LoD were not included).

^a^ppb: parts per billion.

^b^8-h Workplace Exposure Standards (WES) set by Worksafe New Zealand (2020).

^c^8-h Workplace Exposure Standards (TLV-Threshold Limit Value) set by American Conference of Governmental Industrial Hygienists (ACGIH) (2020).

^d^Limit of detection.

^e^ *n* (methanol) = 25.

^f^ *n* (methanol) = 10.

^g^The ratio represents the relative difference in concentration between fumigated and non-fumigated containers with non-fumigated containers selected as the reference category e.g. a ratio of 2 indicates that the concentration of a particular chemical (or the AMV fo all chemicals combined) is twice as high in fumigated compared to non-fumigated containers, while a ratio of 0.7 indicates a 30% reduction in concentration or AMV level

^h^The WES is below the limit of detection.

^i^The ACGIH has not set a TLV for 1,2-dibromoethane.

^j^These chemicals do not have a TWA limit but only a ceiling limit which was used instead

^k^Additive mixture value using the WES and excluding 1,2-dibromoethane.

^l^Additive mixture value using the TLV and excluding 1,2-dibromoethane.

***P < 0.001, **P < 0.01, *P < 0.05.

The detection frequencies for other chemicals were similar in fumigated and non-fumigated containers, except for ammonia, which was detected more frequently in fumigated containers (47.1% versus 10.3%). Methanol was detected in all samples that were analysed for methanol. Formaldehyde was the only chemical exceeding the WES and TLV, in both fumigated and non-fumigated containers. Across all fumigants and other chemicals and excluding 1,2-dibromoethane as the actual number of containers exceeding the WES or TLV is unknown, levels in 10 (22%) containers exceeded the WES, while levels in 31 (67%) containers exceeded the TLV (data not shown).

The overall AMV-WES and AMV-TLV frequently exceeded ‘1’ in both non-fumigated and fumigated containers, although this occurred more often in fumigated containers [52.9% (WES) and 88.2% (TLV) versus 17.2% and 69%; Table 3]. This was most pronounced for AMVs calculated for fumigants only, with fumigated containers exceeding the AMV-WES and AMV-TLV for fumigants 5 (WES) and 12 (TLV) times more frequently than non-fumigated containers (*P* < 0.001; Table 3). Adjusting for cargo, country of origin, container size and number of open vents resulted in only a minor change (AMV-WES for fumigants: ratio 7.5, 95% CI 2.1–27.3; AMV-TLV for fumigants: ratio 7.3, 95% CI 1.6–33.3; Table 4). Also, the exclusion of methanol did not change the results (results not shown).

**Table 4.** Multi-variate regression of AMVs with cargo category, country of origin and other variables from air samples taken upon opening the container door (*n* = 45)

		Fumigants		Other chemicals		Overall	
		AMV-WES^a^	AMV-TLV^b^	AMV-WES	AMV-TLV	AMV-WES	AMV-TLV
Variable	n	Ratio (CI)	Ratio (CI)	Ratio (CI)	Ratio (CI)	Ratio (CI)	Ratio (CI)
Fumigation status							
Not fumigated	28	reference	reference	reference	reference	reference	reference
Fumigated	17	7.5 (2.1–27.3)**	7.3 (1.6–33.3)*	1.4 (0.6–3.1)	1.2 (0.5–2.9)	5.3 (1.8–15.3)**	3.3 (1.0–11.0)*
Cargo							
Miscellaneous	32	reference	reference	Reference	Reference	reference	reference
Cars and metal car parts	4	0.6 (0.1–2.8)	0.8 (0.1–5.3)	0.4 (0.2–1.2)	0.4 (0.1–1.4)	0.5 (0.1–1.7)	0.6 (0.1–2.6)
Tyres	4	0.9 (0.2–4.7)	2.8 (0.4–20.6)	5.1 (1.7–15.2)**	6.2 (1.9–19.9)**	1.1 (0.3–4.6)	2.8 (0.6–13.2)
Unknown	5	2.1 (0.4–10.0)	3.2 (0.5–19.6)	2.8 (1.0–7.7)*	2.8 (0.9–8.2)	3.2 (0.9–11.5)	3.3 (0.8–14.0)
Country							
World excluding Asia	18	reference	reference	Reference	Reference	reference	reference
Asia	20	2.5 (0.9–6.8)	2.5 (0.8–8.0)	1.7 (0.9–3.3)	1.6 (0.8–3.3)	2.7 (1.2–6.2)*	2.6 (1.1–6.6)*
Unknown	7	6.4 (1.7–23.7)**	1.3 (0.3–6.1)	3.4 (1.5–8.0)**	3.6 (1.4–9.0)**	5.2 (1.7–15.4)**	2.6 (0.8–8.8)
Container size							
20 ft	24	reference	reference	Reference	Reference	Reference	reference
40 ft	21	1.3 (0.5–3.0)	2.0 (0.7–5.4)	1.8 (1.0–3.1)*	2.1 (1.1–3.7)*	1.4 (0.7–2.9)	1.8 (0.8–4.0)
Container vents							
2 open vents	23	Reference	reference	Reference	Reference	Reference	reference
No open vents	10	1.6 (0.4–6.2)	2.9 (0.6–14.7)	1.3 (0.5–3.1)	1.3 (0.5–3.4)	1.6 (0.5–5.0)	3.2 (0.9–11.6)
4 open vents	6	0.8 (0.2–3.0)	0.4 (0.1–2.0)	0.6 (0.3–1.4)	0.5 (0.2–1.3)	0.8 (0.3–2.3)	0.6 (0.2–2.0)
Unknown	6	0.2 (0.0–1.1)	0.5 (0.1–3.3)	0.3 (0.1–0.9)*	0.4 (0.1–1.1)	0.2 (0.1–0.9)*	0.4 (0.1–1.9)

^a^Additive mixture value using the WES (8-h workplace exposure standards set by Worksafe New Zealand (2020)) and excluding 1,2-dibromoethane.

^b^Additive mixture value using the TLV (8-h workplace exposure standards (threshold limit value) set by American Conference of Governmental Industrial Hygienists (ACGIH) (2020)) and excluding 1,2-dibromoethane.

*** *p* < 0.001, ** *p* < 0.01, * *p* < 0.05.

Higher AMV levels for other chemicals were observed for the cargo category ‘tyres’ and for larger containers (40 ft); containers from Asia also had higher overall AMV levels (Table 4). Although AMV levels were higher in containers without open vents (when vents were taped over) and lower in containers with four open vents, when compared to two open vents, these differences were not statistically significant (Table 4).

Of the 41 PID measurements collected upon opening containers, 10 had an initial reading of >1000 ppb (lower LoD). For seven containers readings dropped below 1000 ppb within 2 minutes, while for one container, which had been fumigated twice with methyl bromide, it took an hour for levels to drop below 1000 ppb (Figure 1). For this container, an additional sample was taken 33 minutes after opening the doors, showing a drop of methyl bromide from the initial 319 000 to 5826 ppb (1.8%), which remained above both the WES and TLV.

## Discussion

This is the first study that comprehensively assessed airborne fumigants and other chemicals in containers imported into New Zealand. Concentrations in sealed containers were compared with New Zealand (WES) and international (ACGIH TLV) 8-hour exposure limits, which, although not directly applicable to short-term ambient concentrations, provide a conservative comparison. Levels regularly exceeded these limits for one or more chemicals (8% WES; 20% TLV), and additive mixture values (AMV) above ‘1’ were common (AMV-WES 8%, AMV-TLV 26%).

A direct comparison of median levels (based on detectable results only) with results from international studies was not possible as most reported only the frequency of measurements exceeding exposure limits. Two studies reported medians (Knol-de Vos, 2002; Svedberg and Johanson, 2017), but the analytical detection limits were considerably higher than in our study, hampering a valid comparison. Using the Dutch occupational exposure limit (OEL), as also applied in a recent review (European Agency for Safety and Health at Work, 2018), we compared the proportion of exceedances for fumigants and formaldehyde with those reported in 9 international studies (Table S3 available at *Annals of Occupational Hygiene* online). This showed fewer exceedances for chloropicrin and phosphine in our survey. Ethylene oxide exceeded the OEL in 4.5% of containers in our study, similar to the 5.4% reported for Australia, while percentages reported for European countries were generally lower. For methyl bromide and formaldehyde, our results were in the mid-range compared to European countries, and lower than Australia. Despite these differences, which may be due to factors such as cargo, length of travel, differences in measuring methods and changes in fumigation trends over time (Svedberg and Johanson, 2017), this comparison suggests that ambient air concentrations in a significant proportion of containers exceed current exposure standards.

One other study reported AMV values (Svedberg and Johanson, 2017) with ~10% of containers exceeding ‘1’ compared to 7.8% (AMV-WES) and 25.7% (AMV-TLV) in our study (Table 1). However, LoDs and the number of chemicals used in the calculation of the AMVs varied between studies, hampering a valid comparison.

As reported previously (Svedberg and Johanson, 2013; Braconnier and Keller, 2015), and illustrated in Figure 1, ambient concentrations decrease rapidly after opening containers. Also, containers may be ventilated prior to entry, reducing levels further (Svedberg and Johanson, 2013; Hinz *et al.*, 2020). In our smaller survey, all fumigated containers were ventilated for up to 24 h prior to entry, whilst non-fumigated containers had ventilation times ranging from a few minutes up to an hour. Therefore, concentrations in closed containers are unlikely to be a valid estimate of worker exposures as also suggested by the few studies that reported 8-h personal exposures of well-below ambient concentrations (Safe Work Australia, 2011; Svedberg and Johanson, 2013; Hinz *et al.*, 2020). Nonetheless, they represent potential peak exposures that may occur when opening containers. This may be particularly relevant for biosecurity surveillance workers who inspect containers immediately upon opening container doors. Although air extraction units are available, these require container doors to be opened first and are not often used. A recently reported method for pre-ventilation without the need to open container doors (Johanson and Svedberg, 2020) may mitigate this.

Although many chemicals were frequently detected in a large proportion of containers with a wide range of cargo and from a range of countries, rubber products, including tyres, were particularly associated with elevated AMVs (for both fumigants and other chemicals), with exposure ratios ranging from 2.6 to 7.9 (Table 2). Tyres were also associated with elevated AMVs for other chemicals in the smaller study (exposure ratio AMV-WES, 5.1 and AMV-TLV, 6.2, Table 4). To the best of our knowledge, this has not previously reported, although one study found airborne formaldehyde and benzene concentrations above the Dutch OEL in containers carrying rubber products (Luyts and Mück, 2011). As rubber fumes contain hazardous chemicals not measured in our survey (several of which associated with cancers and respiratory symptoms (IARC Working Group, 2012), the reported AMVs are likely an underestimation.

Concentrations of fumigants were positively associated with ‘personal hygiene, beauty and medical products’, ‘stone, ceramics and articles thereof’, and ‘metal and glass’. To the best of our knowledge, this has not previously been reported. AMVs for other chemicals were positively associated with ‘pet food’. Other studies have shown that airborne phosphine more frequently exceeded the Dutch OEL in containers carrying ‘food and feed’ items (Knol-de Vos, 2002; European Agency for Safety and Health at Work, 2018) and one study showed that exposure limits were more frequently exceeded in containers carrying ‘foodstuffs’, mainly due to formaldehyde (Baur *et al.*, 2010). Previous studies have also shown exposure limit exceedances for other cargo types and specific airborne chemicals (shoes/benzene (Baur *et al.*, 2010; Luyts and Mück, 2011; Svedberg and Johanson, 2017); furniture and household items/formaldehyde (Baur *et al.*, 2010); medical devices/ethylene oxide (European Agency for Safety and Health at Work, 2018); and decoration materials/methyl bromide (European Agency for Safety and Health at Work, 2018). Most of these associations were not observed in our study (or could not be studied). However, we found an association with ‘personal hygiene, beauty and medical products’ (Table 2) but further analyses showed that this was attributable to other non-medical products (data not shown). The lack of consistent findings between studies may be due to differences in the sample of containers measured, local and international fumigation practices, fumigation requirements for different countries, air-sampling methodology, categorizations used to combine cargo types, and/or differences in exposure limits used.

The country of origin was not strongly associated with chemical concentrations, although concentrations of other chemicals appeared higher in containers from China (Table 2). A previous study also found that containers from China had the highest frequency of containers with airborne chemicals exceeding chronic and acute exposure limits, but differences between countries were relatively small (Baur *et al.*, 2010). This lack of a clear association with country of origin was also found in another study (Knol-de Vos, 2002), which showed no difference between countries in the proportion of containers exceeding the Dutch OEL.

Our study found that fumigation status, which has not been studied previously, was strongly associated with elevated levels of methyl bromide and phosphine. However, fumigants were also detected in non-fumigated containers, albeit less frequently. Likewise, exceedances of exposure limits were more frequent in fumigated containers, but these also occurred in non-fumigated containers. The presence of fumigants in non-fumigated containers may be due to earlier fumigation of the same container with different cargo; alternatively, some fumigated containers may not have been labelled as fumigated. Regardless, these findings demonstrate the potential for workers to be exposed to fumigants and other harmful chemicals even when handling containers that are not labelled as fumigated, which represent the majority of containers arriving in New Zealand (New Zealand Ministry for Primary Industries, 2019). Methyl bromide and phosphine were the main drivers of elevated AMVs in fumigated containers, while formaldehyde, a carcinogen and dermal sensitizer (Worksafe New Zealand, 2020), was the main driver for elevated AMVs in non-fumigated containers. This again suggests that handling non-fumigated containers may not be without risk.

As reported by others (Svedberg and Johanson, 2013; Braconnier and Keller, 2015; European Agency for Safety and Health at Work, 2018), the current study has shown that ventilation is effective at reducing ambient concentrations of fumigants and off-gassed chemicals. However, it is difficult to assess when safe levels are reached, especially when relying on natural ventilation (Svedberg and Johanson, 2013; Braconnier and Keller, 2015; European Agency for Safety and Health at Work, 2018). Therefore, suitable devices to measure fumigants and off-gassed chemicals are required (Knol-de Vos, 2002; Pedersen *et al.*, 2014; Baur *et al.*, 2015; European Agency for Safety and Health at Work, 2018). A PID monitor is often used, but this does not identify specific chemicals and some of the WES/TLV values are below the LoD of most PIDs. Also, it can provide false positive findings, as volatile compounds with low toxicity (e.g. methanol) are also measured. Other devices are available (gas chromatography-mass spectrometry), but these are often unaffordable, require specialized analytical skills, and often do not provide results in real time. Therefore, in the absence of more affordable, specific and sensitive equipment that does not require specialist training, workers will continue to be at risk of occasional high exposures. Thus, there is a need to establish improved and standardized strategies for the safe inspection and unpacking of shipping containers (Svedberg and Johanson, 2013, 2017; Pedersen *et al.*, 2014; Baur *et al.*, 2015; European Agency for Safety and Health at Work, 2018).

The smaller survey included a higher proportion of fumigated containers than the larger survey. There are several reasons for this. Firstly, the larger study did not include containers fumigated in New Zealand. Secondly, there was selection towards fumigated containers in the smaller study due to preferences of container handlers and management to sample specific containers, which were often fumigated. Thirdly, many of the fumigated containers in the smaller study were fumigated in New Zealand, which, due to strict rules around fumigation, would have increased the number of containers appropriately labelled as fumigated.

Study limitations include the relatively small size of the second survey. It also involved a selective sample; hence, containers were not representative of all containers arriving in New Zealand. Nonetheless, although not representative, the oversampling of fumigated containers ensured sufficient numbers of fumigated containers to meaningfully compare results with non-fumigated containers. For the larger study, containers were selected from one port, which may not be representative all containers arriving in New Zealand i.e. some studies have shown large differences between ports (Knol-de Vos, 2002; Svedberg and Johanson, 2017). In addition, the larger study lacked some information on container characteristics such as number of container vents and the size of the container. Furthermore, the linear regression analyses described in Tables 2 and 4 involve many comparisons, which may risk false positive results. However, we observed considerably more statistical findings than expected based on chance alone i.e. 23 versus 6 (Table 2) and 16 versus 4.5, when defining statistical significance as *P* < 0.05. Therefore, results are unlikely to be due to chance alone.

As air samples were tested only for selected chemicals and the LoD for 1,2-dibromoethane was above the WES, the AMV values are likely underestimated. Similarly, for the second survey we used canister samples that were stored up to 24 or even 48 h, prior to analysis, which may have resulted in decay and subsequent underestimation of the true concentrations. However, validation experiments (see Methods) showed that decay was modest and is therefore unlikely to have significantly affected our results. Nonetheless, as not all tested chemicals were included, we cannot exclude more significant decay for some chemicals. Furthermore, in the larger study, samples were taken at the bottom of the container doors, which may have also resulted in an underestimation of concentrations (Svedberg and Johanson, 2017).

Another limitation of the larger study is that samples were taken in 2011 and may therefore not accurately represent the current situation. However, although some changes may have happened in this industry, it is unlikely that results would be very different, as relevant policies and/or fumigation requirements in New Zealand have not been changed since 2011. Nonetheless, this cannot be objectively verified, and recent changes in import/export patterns due to the 2020/2021 COVID-19 pandemic may have, at least temporarily, resulted in some changes.

In conclusion, this study showed that airborne chemicals in containers arriving in New Zealand frequently exceed exposure limits, both in fumigated and non-fumigated containers. Workers may therefore experience hazardous peak exposures particularly upon opening container doors, also in non-fumigated containers. Although fumigation status and some cargo types and countries of origin were associated with elevated ambient chemical concentrations, results were not always consistent with those reported in other studies and it therefore remains difficult to predict which containers represent the greatest risk.

## Supplementary Material

wxab090_suppl_Supplementary_Tables

## Data Availability

Part of the data underlying this article (the larger survey) were provided by New Zealand Customs Service by permission. Data will be shared on request to the corresponding author with permission of New Zealand Customs Service. The data underlying the smaller survey will be shared on reasonable request to the corresponding author, and upon checking with the committee that provided ethics approval.
