# Supplementary material for: Airborne Fumigants and Residual Chemicals in Shipping Containers Arriving in New Zealand
Source: Ann Work Expo Health. 2021 Oct 18;66(4):481–94. doi: 10.1093/annweh/wxab090 (PMC9030136; doi:10.1093/annweh/wxab090)
Supplement: wxab090_suppl_Supplementary_Tables [file wxab090_suppl_supplementary_tables.docx]

**Airborne fumigants and residual chemicals in shipping containers arriving in New Zealand**

Ruth Hinz^1*^, Andrea ’t Mannetje^1^, Bill Glass^1^, Dave McLean^1^, Jeroen Douwes^1^

^1^ Centre for Public Health Research, Massey University, Wellington, New Zealand

*Author to whom correspondence should be addressed. Tel: +64-4-979-3108, e-mail: r.hinz@massey.ac.nz

**Supplementary Table S1. Frequency table of percentage of detected chemicals in each cargo and country category in the survey on sealed containers (n=490)**

|  | | | | | | | | | | | | | | | **total containers** | **total containers %** | **1,2-Dibromoethane** | **Chloropicrin** | **Ethylene oxide** | **Hydrogen cyanide** | **Phosphine** | **Methyl bromide** | **Fumigants** | **Benzene** | **Formaldehyde** | **Toluene** | **>AMV-WES** | **>AMV-TLV** |
| --- | --- | --- | --- | --- | --- | --- | --- | --- | --- | --- | --- | --- | --- | --- | --- | --- | --- | --- | --- | --- | --- | --- | --- | --- | --- | --- | --- | --- |
| **Total** |  |  |  |  |  |  |  |  |  |  |  |  |  |  | 490 | 100.0 | 2 | 0.2 | 4.7 | 1.8 | 1 | 3.5 | 11.4 | 3.5 | 81 | 31.8 | 7.8 | 25.7 |
| **Cargo** | | | | | | | | | | | | | | |  |  |  |  |  |  |  |  |  |  |  |  |  |  |
| **Food, beverages, tobacco** | | | | | | | | | | | | | | | 77 | 15.7 |  |  |  |  |  |  |  |  |  |  |  |  |
| Beverages, spirits and vinegar | | | | | | | | | | | | | | | 27 | 5.5 | 3.7 | 0 | 0 | 0 | 0 | 0 | 3.7 | 3.7 | 81.5 | 14.8 | 11.1 | 33.3 |
| Preparations of cereals, flour, starch or milk | | | | | | | | | | | | | | | 19 | 3.9 | 0 | 0 | 0 | 0 | 0 | 5.3 | 5.3 | 0 | 84.2 | 10.5 | 5.3 | 5.3 |
| Sugars and sugar confectionery | | | | | | | | | | | | | | | 6 | 1.2 | 16.7 | 0 | 0 | 0 | 0 | 16.7 | 33.3 | 33.3 | 83.3 | 50 | 0 | 33.3 |
| Miscellaneous edible preparations | | | | | | | | | | | | | | | 6 | 1.2 | 0 | 0 | 0 | 0 | 0 | 0 | 0 | 0 | 83.3 | 33.3 | 0 | 16.7 |
| Dairy produce; bird eggs; natural honey; edible products of animal origin | | | | | | | | | | | | | | | 5 | 1.0 | 0 | 0 | 0 | 0 | 0 | 0 | 0 | 0 | 100 | 40 | 0 | 0 |
| Coffee, tea and spices | | | | | | | | | | | | | | | 5 | 1.0 | 20 | 0 | 0 | 0 | 0 | 0 | 20 | 0 | 80 | 20 | 0 | 40 |
| Edible vegetables and certain roots and tubers | | | | | | | | | | | | | | | 3 | 0.6 | 0 | 0 | 0 | 0 | 0 | 0 | 0 | 0 | 100 | 0 | 0 | 0 |
| Cereals | | | | | | | | | | | | | | | 2 | 0.4 | 0 | 0 | 0 | 0 | 0 | 0 | 0 | 0 | 0 | 50 | 0 | 0 |
| Edible fruit and nuts; peel of citrus fruit or melons | | | | | | | | | | | | | | | 1 | 0.2 | 0 | 0 | 0 | 0 | 0 | 0 | 0 | 0 | 100 | 0 | 0 | 0 |
| Cocoa and cocoa preparations | | | | | | | | | | | | | | | 1 | 0.2 | 0 | 0 | 0 | 0 | 0 | 100 | 100 | 0 | 100 | 100 | 0 | 100 |
| Preparations of meat, of fish or of crustaceans, molluscs or other aquatic invertebrates | | | | | | | | | | | | | | | 1 | 0.2 | 0 | 0 | 0 | 0 | 0 | 0 | 0 | 0 | 100 | 100 | 0 | 0 |
| Tobacco and manufactured tobacco substitutes | | | | | | | | | | | | | | | 1 | 0.2 | 0 | 0 | 0 | 0 | 0 | 0 | 0 | 0 | 100 | 0 | 0 | 0 |
| **Paper and paperboard and articles there of** | | | | | | | | | | | | | | | 62 | 12.7 |  |  |  |  |  |  |  |  |  |  |  |  |
| Paper and paperboard; articles of paper pulp, of paper or of paperboard | | | | | | | | | | | | | | | 58 | 11.8 | 0 | 0 | 0 | 0 | 0 | 1.7 | 1.7 | 0 | 82.8 | 5.2 | 0 | 15.5 |
| Printed books, newspapers, pictures and other products of the printing industry | | | | | | | | | | | | | | | 4 | 0.8 | 0 | 0 | 0 | 0 | 0 | 0 | 0 | 0 | 50 | 0 | 0 | 0 |
| **Plastics** | | | | | | | | | | | | | | | 57 | 11.6 |  |  |  |  |  |  |  |  |  |  |  |  |
| Plastics and articles thereof | | | | | | | | | | | | | | | 57 | 11.6 | 1.8 | 0 | 5.3 | 3.5 | 0 | 5.3 | 15.8 | 3.5 | 80.7 | 40.4 | 5.3 | 24.6 |
| **Machinery and equipment including appliances and electronics** | | | | | | | | | | | | | | | 49 | 10.0 |  |  |  |  |  |  |  |  |  |  |  |  |
| Nuclear reactors, boilers, machinery and mechanical appliances; parts thereof | | | | | | | | | | | | | | | 23 | 4.7 | 0 | 0 | 4.3 | 0 | 0 | 4.3 | 8.7 | 4.3 | 78.3 | 39.1 | 4.3 | 21.7 |
| Electrical machinery and equipment and parts thereof; sound recorders, TV | | | | | | | | | | | | | | | 22 | 4.5 | 0 | 0 | 0 | 0 | 0 | 0 | 0 | 0 | 100 | 40.9 | 4.5 | 31.8 |
| Optical, photographic, cinematographic, measuring, checking, precision, medical or surgical | | | | | | | | | | | | | | | 4 | 0.8 | 0 | 0 | 0 | 0 | 0 | 0 | 0 | 0 | 75 | 25 | 0 | 25 |
| **Personal hygiene, beauty and medical products** | | | | | | | | | | | | | | | 43 | 8.8 |  |  |  |  |  |  |  |  |  |  |  |  |
| Essential oils and resinoids; perfumery, cosmetic or toilet preparations | | | | | | | | | | | | | | | 22 | 4.5 | 0 | 0 | 9.1 | 9.1 | 0 | 9.1 | 27.3 | 4.5 | 86.4 | 27.3 | 9.1 | 50 |
| Pharmaceutical products | | | | | | | | | | | | | | | 14 | 2.9 | 0 | 0 | 7.1 | 7.1 | 0 | 0 | 14.3 | 0 | 78.6 | 28.6 | 7.1 | 7.1 |
| Soap, organic surface-active agents, washing preparations, lubricating preparations, artificial | | | | | | | | | | | | | | | 6 | 1.2 | 0 | 0 | 0 | 33.3 | 0 | 0 | 33.3 | 16.7 | 83.3 | 33.3 | 16.7 | 66.7 |
| Albuminoidal substances; modified starches; glues; enzymes | | | | | | | | | | | | | | | 1 | 0.2 | 0 | 0 | 0 | 0 | 0 | 0 | 0 | 0 | 0 | 0 | 0 | 0 |
| **Chemicals** | | | | | | | | | | | | | | | 39 | 8.0 |  |  |  |  |  |  |  |  |  |  |  |  |
| Miscellaneous chemical products | | | | | | | | | | | | | | | 16 | 3.3 | 6.3 | 0 | 0 | 6.3 | 0 | 0 | 12.5 | 6.3 | 87.5 | 37.5 | 6.3 | 31.3 |
| Tanning or dyeing extracts; tannins and their derivatives; dyes, pigments and other | | | | | | | | | | | | | | | 12 | 2.5 | 0 | 0 | 8.3 | 0 | 0 | 0 | 8.3 | 0 | 83.3 | 50 | 8.3 | 16.7 |
| Salt; sulphur; earths and stone; plastering materials, lime and cement | | | | | | | | | | | | | | | 3 | 0.6 | 0 | 0 | 0 | 0 | 0 | 0 | 0 | 0 | 66.7 | 0 | 0 | 0 |
| Organic chemicals | | | | | | | | | | | | | | | 3 | 0.6 | 0 | 0 | 0 | 0 | 0 | 0 | 0 | 0 | 100 | 0 | 33.3 | 33.3 |
| Mineral fuels, mineral oils and product | | | | | | | | | | | | | | | 2 | 0.4 | 0 | 0 | 50 | 0 | 0 | 0 | 50 | 0 | 100 | 0 | 50 | 50 |
| Inorganic chemicals; organic or inorganic compounds of precious metals, of rare-earth metals, | | | | | | | | | | | | | | | 2 | 0.4 | 0 | 0 | 50 | 0 | 0 | 0 | 50 | 0 | 50 | 0 | 50 | 0 |
| Photographic or cinematographic goods | | | | | | | | | | | | | | | 1 | 0.2 | 100 | 0 | 0 | 0 | 0 | 0 | 100 | 0 | 100 | 100 | 0 | 0 |
| **Metal and glass** | | | | | | | | | | | | | | | 36 | 7.3 |  |  |  |  |  |  |  |  |  |  |  |  |
| Articles of iron or steel | | | | | | | | | | | | | | | 15 | 3.1 | 6.7 | 0 | 13.3 | 0 | 0 | 0 | 20 | 6.7 | 53.3 | 80 | 20 | 20 |
| Glass and glassware | | | | | | | | | | | | | | | 9 | 1.8 | 11.1 | 0 | 0 | 0 | 0 | 11.1 | 11.1 | 0 | 55.6 | 33.3 | 11.1 | 22.2 |
| Miscellaneous articles of base metal | | | | | | | | | | | | | | | 5 | 1.0 | 0 | 0 | 0 | 0 | 0 | 0 | 0 | 0 | 80 | 20 | 0 | 0 |
| Tools, implements, cutlery, spoons and forks, of base metal; | | | | | | | | | | | | | | | 4 | 0.8 | 0 | 0 | 0 | 0 | 0 | 0 | 0 | 0 | 25 | 0 | 0 | 0 |
| Aluminium and articles thereof | | | | | | | | | | | | | | | 2 | 0.4 | 0 | 0 | 100 | 0 | 0 | 50 | 100 | 0 | 50 | 100 | 100 | 100 |
| Iron and steel | | | | | | | | | | | | | | | 1 | 0.2 | 0 | 0 | 0 | 0 | 0 | 0 | 0 | 0 | 100 | 100 | 0 | 0 |
| **Man-made fibre articles there of** | | | | | | | | | | | | | | | 34 | 6.9 |  |  |  |  |  |  |  |  |  |  |  |  |
| Furniture; bedding, mattresses, mattress supports, cushions and similar stuffed furnishings | | | | | | | | | | | | | | | 16 | 3.3 | 0 | 0 | 6.3 | 0 | 0 | 6.3 | 12.5 | 0 | 93.8 | 31.3 | 6.3 | 25 |
| Carpets and other textile floor coverings | | | | | | | | | | | | | | | 5 | 1.0 | 0 | 0 | 0 | 0 | 0 | 0 | 0 | 0 | 80 | 20 | 0 | 0 |
| Man-made filaments; strip and the like of man-made textile materials | | | | | | | | | | | | | | | 3 | 0.6 | 0 | 0 | 0 | 0 | 0 | 0 | 0 | 0 | 100 | 0 | 0 | 0 |
| Other made-up textile articles; sets; worn clothing and worn textile articles; rags | | | | | | | | | | | | | | | 3 | 0.6 | 0 | 0 | 0 | 0 | 0 | 0 | 0 | 0 | 100 | 33.3 | 0 | 33.3 |
| Footwear, gaiters and the like; parts of such articles | | | | | | | | | | | | | | | 3 | 0.6 | 0 | 0 | 0 | 0 | 0 | 0 | 0 | 0 | 100 | 33.3 | 0 | 0 |
| Man-made staple fibres | | | | | | | | | | | | | | | 2 | 0.4 | 0 | 0 | 0 | 0 | 0 | 0 | 0 | 0 | 0 | 0 | 0 | 0 |
| Impregnated, coated, covered or laminated textile fabrics | | | | | | | | | | | | | | | 1 | 0.2 | 0 | 0 | 0 | 0 | 0 | 0 | 0 | 0 | 0 | 0 | 0 | 0 |
| Articles of apparel and clothing accessories, knitted or crocheted | | | | | | | | | | | | | | | 1 | 0.2 | 0 | 0 | 0 | 0 | 0 | 0 | 0 | 0 | 100 | 0 | 0 | 0 |
| **Miscellaneous** | | | | | | | | | | | | | | | 32 | 6.5 |  |  |  |  |  |  |  |  |  |  |  |  |
| LCL (multiple commodities) | | | | | | | | | | | | | | | 11 | 2.2 | 0 | 0 | 0 | 9.1 | 0 | 0 | 9.1 | 9.1 | 54.5 | 18.2 | 0 | 27.3 |
| Miscellaneous New Zealand Provisions | | | | | | | | | | | | | | | 9 | 1.8 | 0 | 0 | 0 | 0 | 0 | 0 | 0 | 0 | 77.8 | 66.7 | 0 | 33.3 |
| Toys, games and sports requisites; parts and accessories thereof | | | | | | | | | | | | | | | 7 | 1.4 | 0 | 0 | 0 | 0 | 0 | 0 | 0 | 0 | 85.7 | 42.9 | 14.3 | 28.6 |
| Manufactures of straw, of esparto or of other plaiting materials; basketware and wickerwork | | | | | | | | | | | | | | | 2 | 0.4 | 0 | 50 | 0 | 0 | 0 | 0 | 50 | 50 | 100 | 100 | 50 | 100 |
| Articles of leather and animal gut | | | | | | | | | | | | | | | 1 | 0.2 | 0 | 0 | 0 | 0 | 0 | 0 | 0 | 0 | 100 | 0 | 0 | 0 |
| Miscellaneous manufactured articles | | | | | | | | | | | | | | | 1 | 0.2 | 0 | 0 | 0 | 0 | 0 | 0 | 0 | 0 | 100 | 0 | 0 | 0 |
| Umbrellas, sun umbrellas, walking-sticks, seat-sticks, whips, riding-crops | | | | | | | | | | | | | | | 1 | 0.2 | 0 | 0 | 0 | 0 | 0 | 0 | 0 | 0 | 100 | 100 | 0 | 100 |
| **Wood and articles there of** | | | | | | | | | | | | | | | 18 | 3.7 |  |  |  |  |  |  |  |  |  |  |  |  |
| Wood and articles of wood; wood charcoal | | | | | | | | | | | | | | | 18 | 3.7 | 0 | 0 | 5.6 | 0 | 5.6 | 0 | 5.6 | 5.6 | 77.8 | 55.6 | 5.6 | 27.8 |
| **Stone, ceramics and articles there of** | | | | | | | | | | | | | | | 14 | 2.9 |  |  |  |  |  |  |  |  |  |  |  |  |
| Articles of stone, plaster, cement, asbestos, mica or similar materials | | | | | | | | | | | | | | | 11 | 2.2 | 0 | 0 | 18.2 | 0 | 0 | 36.4 | 36.4 | 9.1 | 90.9 | 36.4 | 18.2 | 45.5 |
| Ceramic products | | | | | | | | | | | | | | | 3 | 0.6 | 0 | 0 | 0 | 0 | 0 | 0 | 0 | 0 | 66.7 | 33.3 | 0 | 0 |
| **Rubber products inclusive tyres** | | | | | | | | | | | | | | | 13 | 2.7 |  |  |  |  |  |  |  |  |  |  |  |  |
| Rubber and articles thereof | | | | | | | | | | | | | | | 13 | 2.7 | 15.4 | 0 | 30.8 | 0 | 30.8 | 0 | 46.2 | 23.1 | 100 | 69.2 | 53.8 | 69.2 |
| **Pet food** | | | | | | | | | | | | | | | 9 | 1.8 |  |  |  |  |  |  |  |  |  |  |  |  |
| Residues and waste from the food industries; prepared animal fodder | | | | | | | | | | | | | | | 9 | 1.8 | 0 | 0 | 0 | 0 | 0 | 0 | 0 | 0 | 100 | 0 | 0 | 77.8 |
| **Vehicles other than railway or tramway rolling stock, and parts** | | | | | | | | | | | | | | | 7 | 1.4 |  |  |  |  |  |  |  |  |  |  |  |  |
| Vehicles other than railway or tramway rolling stock, and parts | | | | | | | | | | | | | | | 7 | 1.4 | 0 | 0 | 14.3 | 0 | 0 | 0 | 14.3 | 0 | 71.4 | 57.1 | 14.3 | 0 |
| **Country** | | | | | | | | | | | | | | |  |  |  |  |  |  |  |  |  |  |  |  |  |  |
| **Total** | | | | | | | | | | | | | | | **490** | **100** | **2.0** | **0.2** | **4.7** | **1.8** | **1.0** | **3.5** | **11.4** | **3.5** | **81.0** | **31.8** | **7.8** | **25.7** |
| Australia | | | | | | | | | | | | | | | 283 | 57.8 | 1.0 | 0.0 | 2.2 | 1.2 | 0.0 | 2.0 | 6.1 | 1.2 | 45.9 | 16.3 | 3.1 | 11.0 |
| China | | | | | | | | | | | | | | | 74 | 15.1 | 0.8 | 0.0 | 1.2 | 0.0 | 0.2 | 0.6 | 1.8 | 1.0 | 12.7 | 6.5 | 2.2 | 5.9 |
| North America | | | | | | | | | | | | | | | 56 | 11.4 | 0.2 | 0.0 | 0.2 | 0.2 | 0.0 | 0.2 | 0.8 | 0.6 | 9.2 | 3.3 | 0.4 | 4.1 |
| Other Asia countries | | | | | | | | | | | | | | | 33 | 6.7 | 0.0 | 0.2 | 0.0 | 0.2 | 0.4 | 0.2 | 1.0 | 0.4 | 5.5 | 3.7 | 0.6 | 2.0 |
| Europe | | | | | | | | | | | | | | | 21 | 4.3 | 0.0 | 0.0 | 0.6 | 0.0 | 0.2 | 0.2 | 0.8 | 0.0 | 3.5 | 1.4 | 1.0 | 1.4 |
| Other regions | | | | | | | | | | | | | | | 23 | 4.7 | 0.0 | 0.0 | 0.4 | 0.2 | 0.2 | 0.2 | 0.8 | 0.2 | 4.3 | 0.6 | 0.4 | 1.2 |

**Supplementary Table S2. Cargo and country of origin of containers by fumigation status in containers upon opening of container doors (n=46)**

| Cargo | non-fumigated containers  (n=29) | fumigated containers  (n=17) | Country | non-fumigated  containers  (n=29) | fumigated containers  (n=17) |
| --- | --- | --- | --- | --- | --- |
| unknown | 6 | 0 | China | 7 | 3 |
| food & tobacco | 5 | 6 | unknown | 6 | 1 |
| textiles | 5 | 0 | Chile | 4 | 0 |
| household items, hardware | 5 | 1 | Asia unknown | 2 | 0 |
| eucalyptus pulp | 4 | 0 | Brazil | 2 | 0 |
| miscellaneous | 2 | 0 | Zimbabwe | 2 | 0 |
| cars and metal car parts | 1 | 3 | Thailand | 1 | 2 |
| tyres | 1 | 3 | Australia | 1 | 1 |
| wood & wooden furniture | 0 | 4 | Pakistan | 1 | 0 |
|  |  |  | Belgium | 1 | 0 |
|  |  |  | UK | 1 | 0 |
|  |  |  | Honduras | 1 | 0 |
|  |  |  | Malawi | 0 | 3 |
|  |  |  | Indonesia | 0 | 4 |
|  |  |  | Malaysia | 0 | 1 |
|  |  |  | USA | 0 | 1 |
|  |  |  | Ghana | 0 | 1 |

**Supplementary Table S3. Comparison of chemicals detected in containers above the Dutch occupational exposure limit (OEL) in the survey on sealed containers with previous studies. Table adapted from (European Agency for Safety and Health at Work, 2018)**

| **Study** | **n (containers)** | **Percentage of containers with chemical level > Dutch OEL (highest detected concentration in ppm)^*^** | | | | |
| --- | --- | --- | --- | --- | --- | --- |
|  |  | **Chloropicrin** | **Ethylene oxide** | **Phosphine** | **Methyl bromide** | **Formaldehyde** |
| **Dutch OEL (ppm)** |  | **0.1** | **0.5** | **0.1** | **0.25** | **0.1** |
| Current study - Tauranga, New Zealand 2011 (New Zealand Customs Service, 2012) | 490 | 0.0 (0.05) | 4.5 (9.7) | 0.2 (0.14) | 1.8 (49.9) | 17.9 (6.6) |
|  |  |  |  |  |  |  |
| Sweden, 2013 (Svedberg and Johanson, 2017) | 249 | n.d. | 0.7 (1.7) | 0.4 | n.d. | 3.6 (2) |
| Australia, 2012 (Safe Work Australia, 2011) | 76 | 5.3 (1.6) | 0 | 1.3 (0.15) | 21.1 (4.4) | 21.1 (2) |
| Europe, 2011 (Mück and Stock, 2012) | 123,439 | 0.01 (26) | n.d. | 1.5 (329) | 0.4 | 3.7 (38) |
| Belgium and the Netherlands, 2010 (Luyts and Mück, 2011) | 42,888 | 0.2 (26) | n.d. | 0.9 (368) | 0.7 (88) | 2.6 (40) |
| Italy, 2004-2010 (Tortarolo, 2011) | 5414/1362/9,482^a^ | n.d. | n.d. | 47.2 (680) | > 13.8 (1,380)^b^ | 4.7 |
| Australia, 2007-2008 (Frost, 2010) | 14,943 | 18.0 | 5.4 | 3.5 | 13.0 | 31.6 |
| Hamburg, Germany, 2006 (Baur et al., 2010) | 2,113 | 1.3 | 0.9 | 0.9 | 7.1 | 30.9 |
| The Netherlands, 2003-2006 (de Groot, 2007) | 277 | 2.4 | n.d. | 2.9 | 13.4 | n.d. |
| Rotterdam, the Netherlands, 2002 (Knol-de Vos, 2002) | 303 | n.d. | n.d. | 2.0 (> 20) | 3.4 (90) | 1.3 (13.4) |

^*^ 1,2 dichloroethane was not included as it was not measured in the NZ survey. Additionally, the European Agency for Safety and Health at Work noted that both the Australian studies found 1,2-dibromoethane in several containers; in particular, the larger survey found that 26.1 % of the containers had 1,2-dibromoethane levels exceeding the OEL. In the NZ survey > 2% of containers exceeded the OEL. The exact value is unknown as the OEL is below the Limit of Detection.

n.d. not determined.

^a^ n=5414 for phosphine, n=1362 for methyl bromide, and n=9482 for formaldehyde

^b^ 13.8 % >1 ppm.
